# Supplementary material for: DELAY OF GERMINATION1 requires PP2C phosphatases of the ABA signalling pathway to control seed dormancy
Source: Nat Commun. 2017 Jul 13;8:72. doi: 10.1038/s41467-017-00113-6 (PMC5509711; doi:10.1038/s41467-017-00113-6)
Supplement: Supplementary file 1 — Supplementary Information [file 41467_2017_113_MOESM1_ESM.pdf]

Title of file for HTML: Supplementary Information
Description: Supplementary Figures, Supplementary Tables and Supplementary References

Title of file for HTML: Supplementary Data 1
Description: Perseus output table of identified and quantified proteins by MS for pull-down assays (Proteins_groups matrix)

Title of file for HTML: Peer Review File
Description:

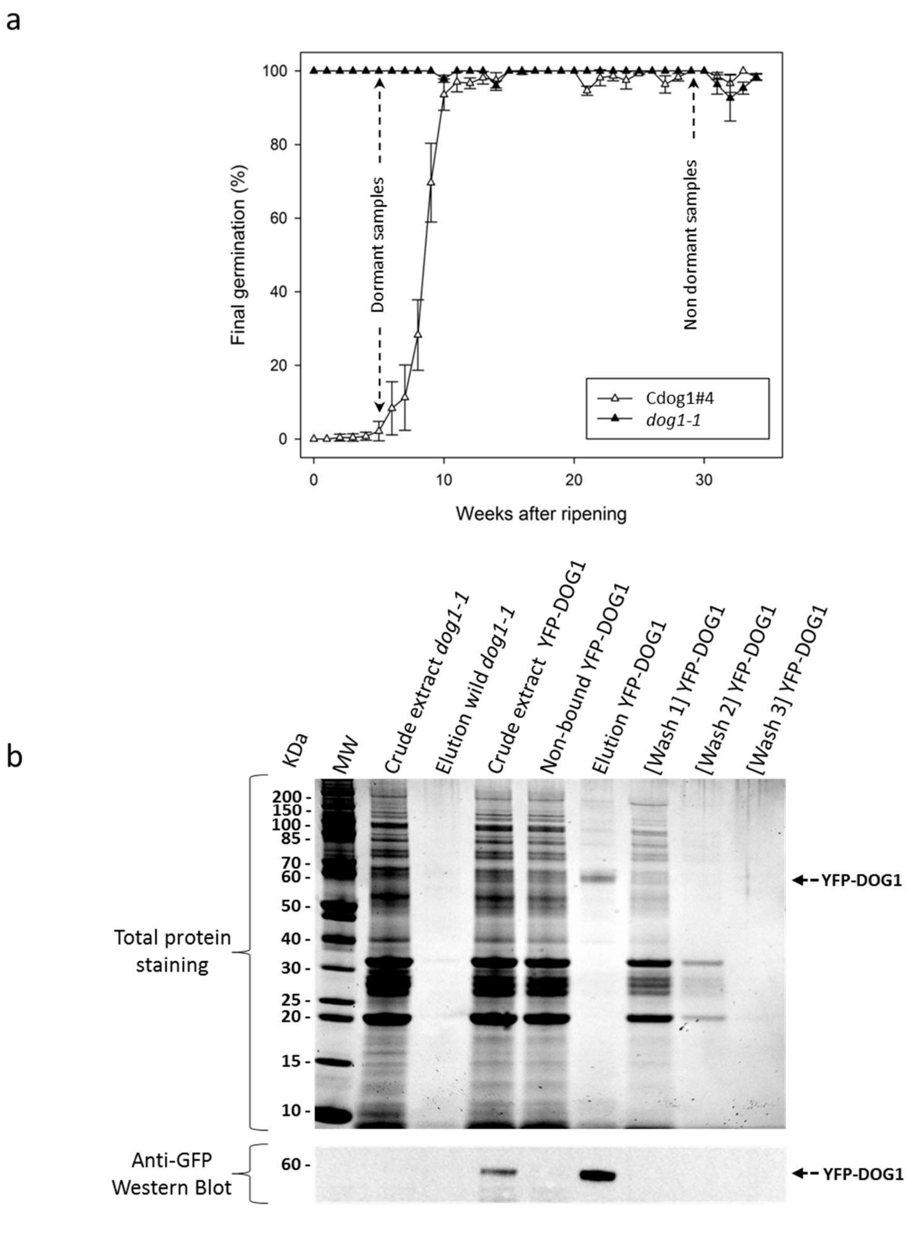

Supplementary Figure 1. After-ripening kinetics of *dog1-1* and the complemented line Cdog1 #4 seeds used for pull-down and SDS-PAGE analysis of the pull-down procedure. (**a**) Germination after different periods of dry storage of seeds from the *dog1-1* mutant and the complementation line Cdog1#4 that were used for pull-down. The germination of the samples at the time of the pull-down is indicated with arrows. (**b**) Gel analysis of the pull-down procedure. YFP-DOG1 was immuno-purified using anti-GFP agarose coated beads (chromotek GFP trap-A). Extract from the *dog1-1* mutant was used as background control. An oriole fluorescent total staining for total protein is shown in the top. The most intense protein signal in the YFP-DOG1 pull-down elution fraction showed the expected size for the YFP-DOG1 fusion protein while no corresponding signal was visible in the background (*dog1-1*) elution fraction. The identity of YFP-DOG1 was confirmed by Western blotting analysis of the same samples using GFP antibody (shown at the bottom) and mass spectrometry (Supplementary Dataset 1).

a

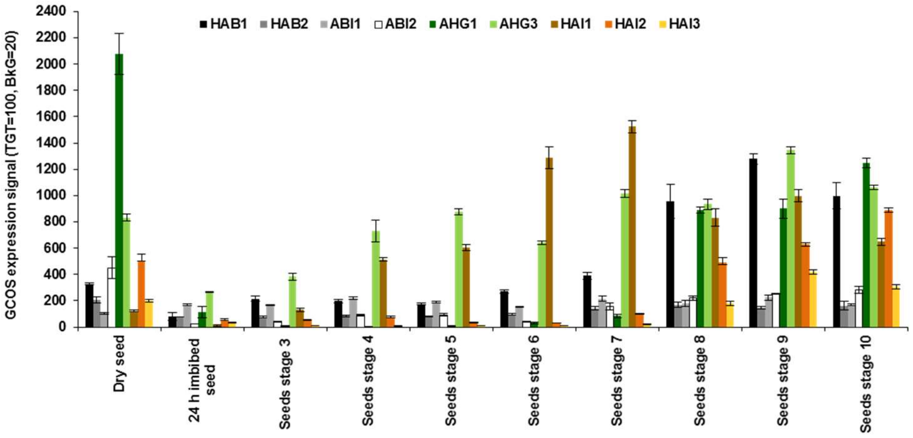

b

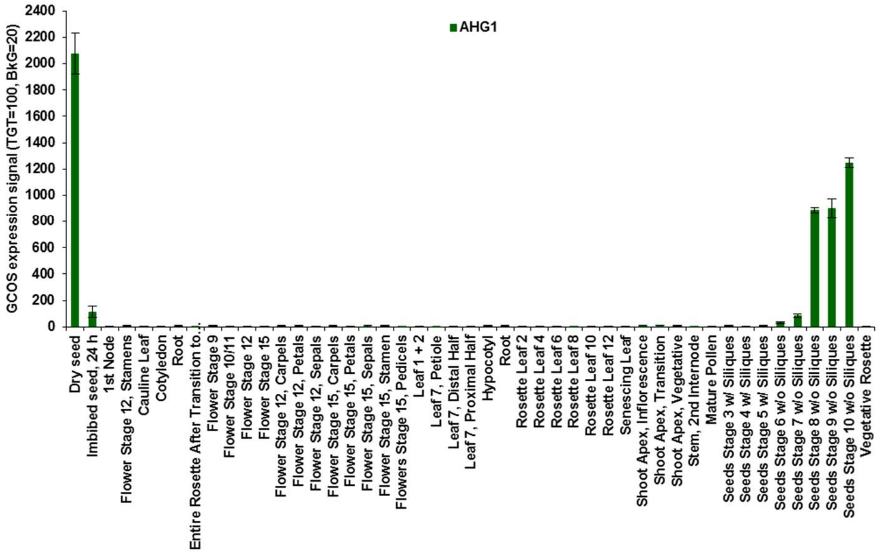

Supplementary Figure 2. Transcript levels of clade A PP2C. (**a**) Relative mRNA accumulation of the nine clade A PP2Cs during seed development and in dry and imbibed seeds. (**b**) Relative mRNA accumulation of *AHG1* during diverse plant developmental stages. Shown are normalized relative expression levels using the GeneChip Operating Software (CGOS) methods with a global scaling to target signal (TGT) =100 and a background (BkG) of 20. The graphs are based on publicly available data (http://bar.utoronto.ca/efp/cgi-bin/efpWeb.cgi).

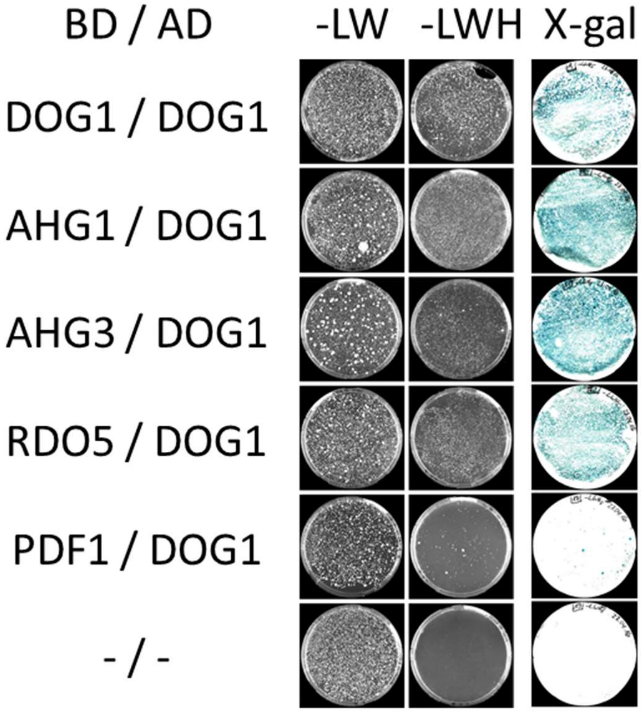

Supplementary Figure 3. Confirmation of interaction between DOG1 and the phosphatases by yeast two-hybrid assay. The left panel shows yeast growth on selective medium, the right panel shows β-galactosidase activity. Co-transformation with empty vectors is shown as negative control. BD, fusion with GAL4 DNA binding domain; AD, fusion with GAL4 activation domain; -LW, dropout media without leucine and tryptophan; -LWH, dropout media without leucine, tryptophan and histidine; -, empty vector.

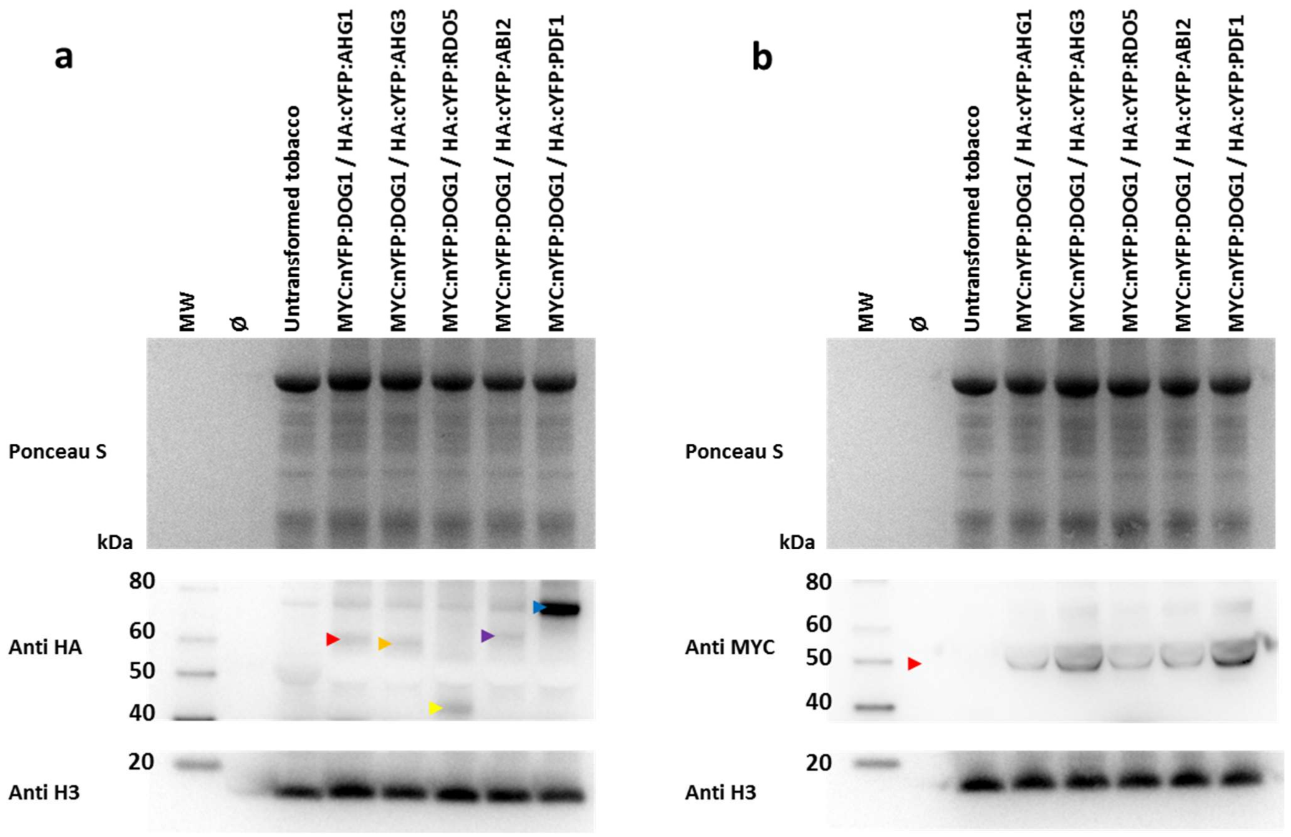

Supplementary Figure 4. Accumulation of split-YFP fusion proteins in transfected tobacco leaves. Total proteins were extracted from the same leaves used for BiFC assays (Fig. 3). 30 µg of protein was separated and blotted against HA-tag antibody (**a**) or MYC-tag antibody (**b**). Signal for the expected size of AHG1 (56,7 kDA), AHG3 (54,1 kDa), RDO5 (42,5 kDa), ABI2 (57 kDa), and PDF1 (76,3 kDa) fusion proteins are marked by a red, orange, yellow, purple, and blue arrow respectively (**a**). Signal for the expected size of DOG1 (49,5 kDa) fusion protein is marked by a red arrow (**b**). After stripping, the same membrane was blotted with H3 antibody as loading control.

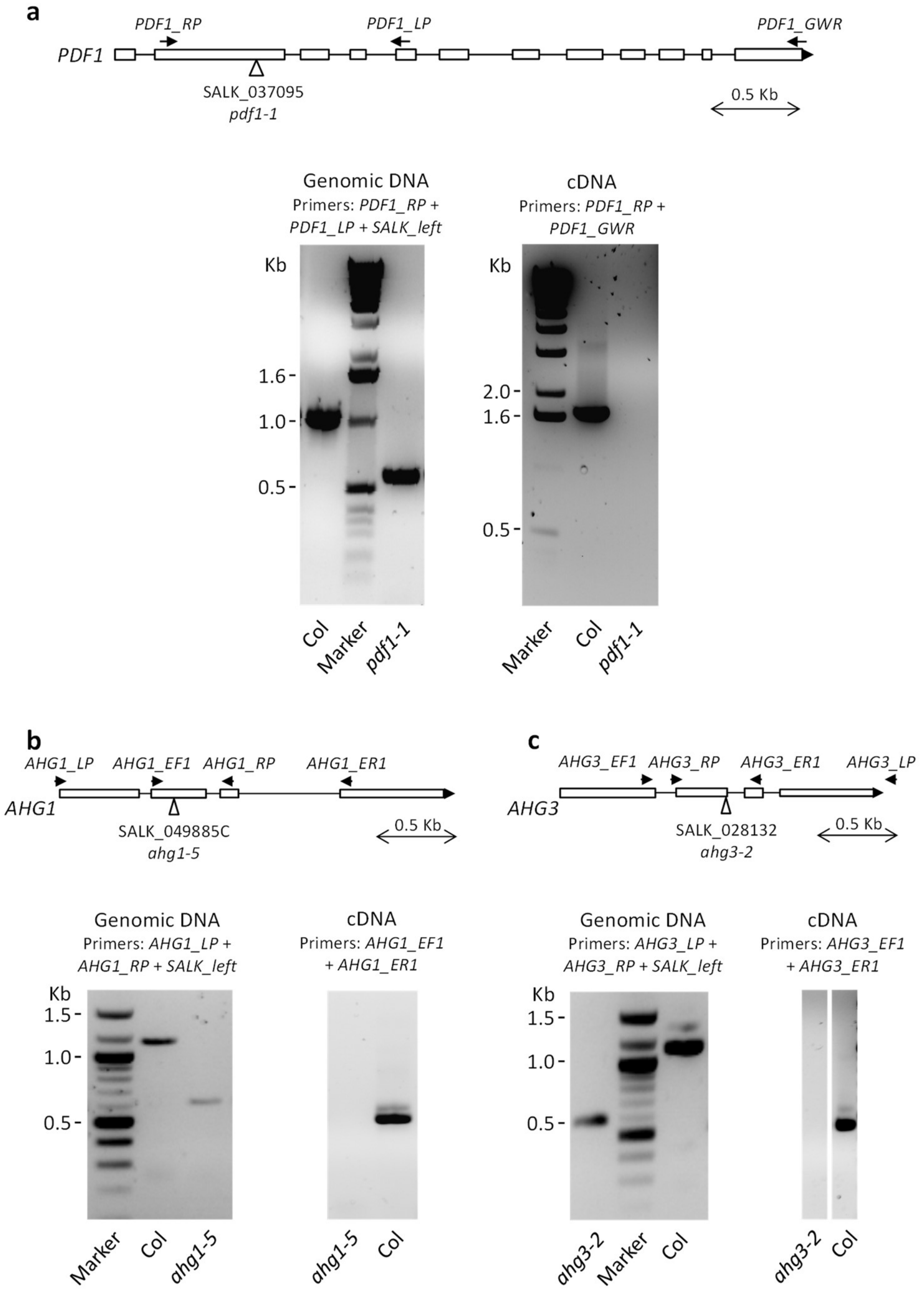

Supplementary Figure 5. Characterization of insertion mutants. Schematic presentation of the *PDF1* gene (**a**), the *AHG1* gene (**b**) and the *AHG3* gene (**c**) are shown at the top of each panel. Boxes indicate exons and lines introns. The location of the insertion is indicated with a triangle. The locations of the primers used for detection of the insert and transcript are indicated with arrows above the gene model. The left bottom of each panel shows PCR analyses for the presence of the T-DNA insertion in the insertion mutants of *PDF1* (**a**), *AHG1* (**b**), and *AHG3* (**c**). The right bottom of each panel shows RT-PCR analyses of *PDF1* (**a**), *AHG1* (**b**), and *AHG3* (**c**) transcripts from seeds of wild-type Col and the respective T-DNA insertion mutants.

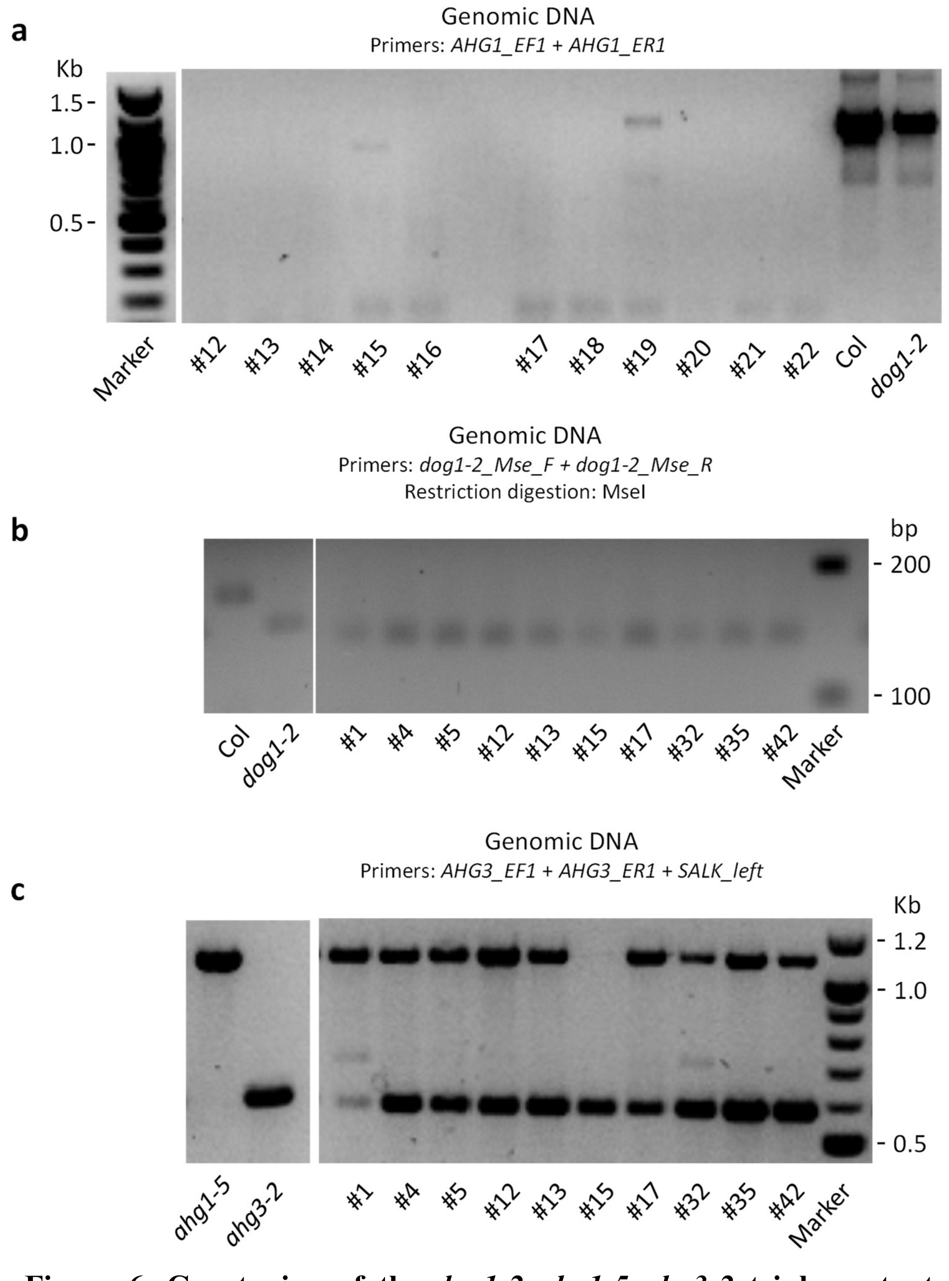

Supplementary Figure 6. Genotyping of the *dog1-2 ahg1-5 ahg3-2* triple mutant. Analysis of F2 individuals from a cross between the double mutants *dog1-2 ahg1-5* and *dog1-2 ahg3-2*. This F2 population is homozygous for the *dog1-2* mutation and segregates for *ahg1-5* and *ahg3-2*. F2 seeds were preselected by their inability to germinate after 1 week imbibition. The selected seeds were subsequently germinated by imbibition on GA_{4+7}. (**a**) Detection of the T-DNA insertion in *ahg1-5* in F2 plants by PCR. Shown are 11 F2 plants out of 88 that had been tested. All 11 plants are homozygous for the *ahg1-5* mutation. Col wildtype and the *dog1-2* mutants were used as controls containing the wild-type *AHG1* gene. (**b**) Detection of the *dog1-2* mutation using a dCAPS marker. The amplified fragment is cut with MseI in the *dog1-2* mutant but not in wild-type Col. All tested F2 plants were homozygous for the *dog1-2* mutation. (**c**) Detection of the T-DNA insertion in *AHG3* by PCR using three primers. The top fragment indicates presence of the wild-type *AHG3* gene and the lower band presence of the T-DNA insertion. The *ahg1-5* mutant was used as wild-type control (shown in the left). Plant #15 was selected as triple mutant and used for phenotypic analyses, the other F2 plants were heterozygous. A subset of the analysed F2 plants is shown on the gels.

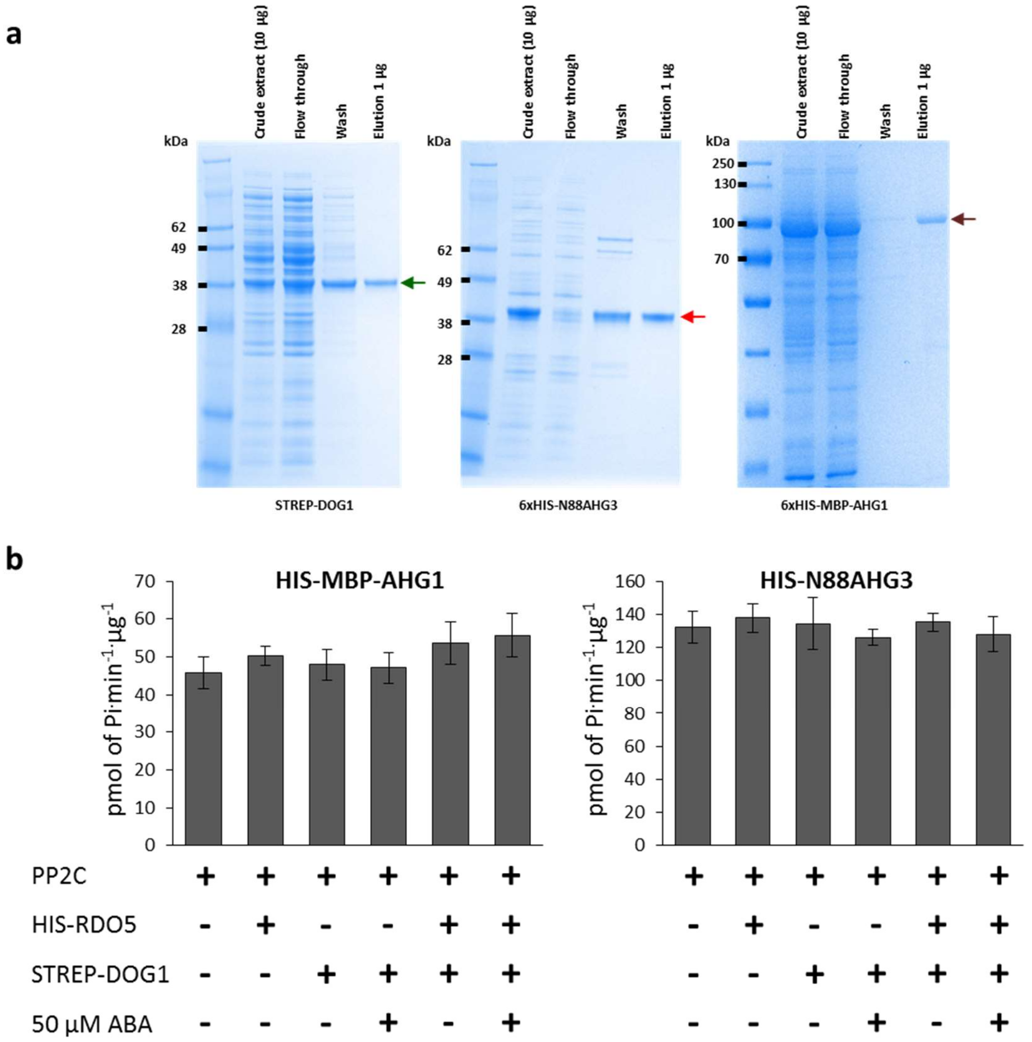

Supplementary Figure 7. *In vitro* phosphatase assays on purified recombinant proteins. (**a**) Gel analysis of recombinant protein purification. All recombinant proteins used in this study were purified to full homogeneity. Coloured arrows indicate the position of a protein band corresponding to the recombinant protein. Purification of full length 6xHIS-RDO5 as well as its pseudophosphatase activity was already described[1]. (**b**) *In vitro* phosphatase activity assays. The effect of DOG1 on AHG1 and AHG3 phosphatase activities was tested by pre-incubation of the PP2C with DOG1 for 20 min at a stoichiometric ratio PP2C:DOG1 of 1:10 before starting the phosphatase reaction by addition of the substrate. The influence of the pseudophosphatase RDO5 alone or in presence of DOG1 on AHG1 and AHG3 activities was tested similarly (stoichiometric ratio RDO5:PP2C of 1:1 and AHG1:RDO5:DOG1 of 1:1:10). When mentioned, the effect of ABA in these experimental setups was tested by addition of ABA at a final concentration of 50µM from the startof the pre-incubation. None of the tested conditions led to significant changes in AHG1 or AHG3 activities. Shown are mean and standard deviation of three replicates.

a

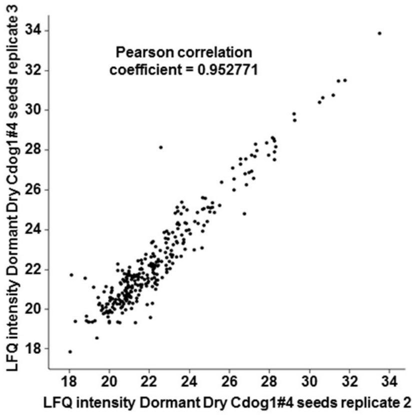

b

	LFQ intensity dormant dry *dog1-1* seeds replicate 1	LFQ intensity dormant dry *dog1-1* seeds replicate 2
LFQ intensity dormant dry *dog1-1* seeds replicate 2	0.766121	
LFQ intensity dormant dry *dog1-1* seeds replicate 3	0.8437681	0.8636715

	LFQ intensity dormant dry Cdog1#4 seeds replicate 1	LFQ intensity dormant dry Cdog1#4 seeds replicate 2
LFQ intensity dormant dry Cdog1#4 seeds replicate 2	0.9227797	
LFQ intensity dormant dry Cdog1#4 seeds replicate 3	0.9301488	0.9527711

	LFQ intensity dormant imbibed *dog1-1* seeds replicate 1	LFQ intensity dormant imbibed *dog1-1* seeds replicate 2
LFQ intensity dormant imbibed *dog1-1* seeds replicate 2	0.9023336	
LFQ intensity dormant imbibed *dog1-1* seeds replicate 3	0.8152048	0.8870851

	LFQ intensity dormant imbibed Cdog1#4 seeds replicate 1	LFQ intensity dormant imbibed Cdog1#4 seeds replicate 2
LFQ intensity dormant imbibed Cdog1#4 seeds replicate 2	0.9353895	
LFQ intensity dormant imbibed Cdog1#4 seeds replicate 3	0.9304259	0.9400388

	LFQ intensity non dormant dry *dog1-1* seeds replicate 1	LFQ intensity non dormant dry *dog1-1* seeds replicate 2
LFQ intensity non dormant dry *dog1-1* seeds replicate 2	0.9219559	
LFQ intensity non dormant dry *dog1-1* seeds replicate 3	0.9258727	0.866001

	LFQ intensity non dormant dry Cdog1#4 seeds replicate 1	LFQ intensity non dormant dry Cdog1#4 seeds replicate 2
LFQ intensity non dormant dry Cdog1#4 seeds replicate 2	0.8328074	
LFQ intensity non dormant dry Cdog1#4 seeds replicate 3	0.83983	0.8971636

	LFQ intensity non dormant imbibed *dog1-1* seeds replicate 1	LFQ intensity non dormant imbibed *dog1-1* seeds replicate 2
LFQ intensity non dormant imbibed *dog1-1* seeds replicate 2	0.9029553	
LFQ intensity non dormant imbibed *dog1-1* seeds replicate 3	0.7336717	0.7520942

	LFQ intensity non dormant imbibed Cdog1#4 seeds replicate 1	LFQ intensity non dormant imbibed Cdog1#4 seeds replicate 2
LFQ intensity dormant imbibed Cdog1#4 seeds replicate 2	0.9187418	
LFQ intensity dormant imbibed Cdog1#4 seeds replicate 3	0.858981	0.9446475

Supplementary Figure 8. Quality analysis of the pulldown replicates. (**a**) Example of Label Free Quantitative (LFQ) intensities from two replicates plotted against each other. (**b**) Table summarizing the Pearson correlation coefficients for all analysed replicates.

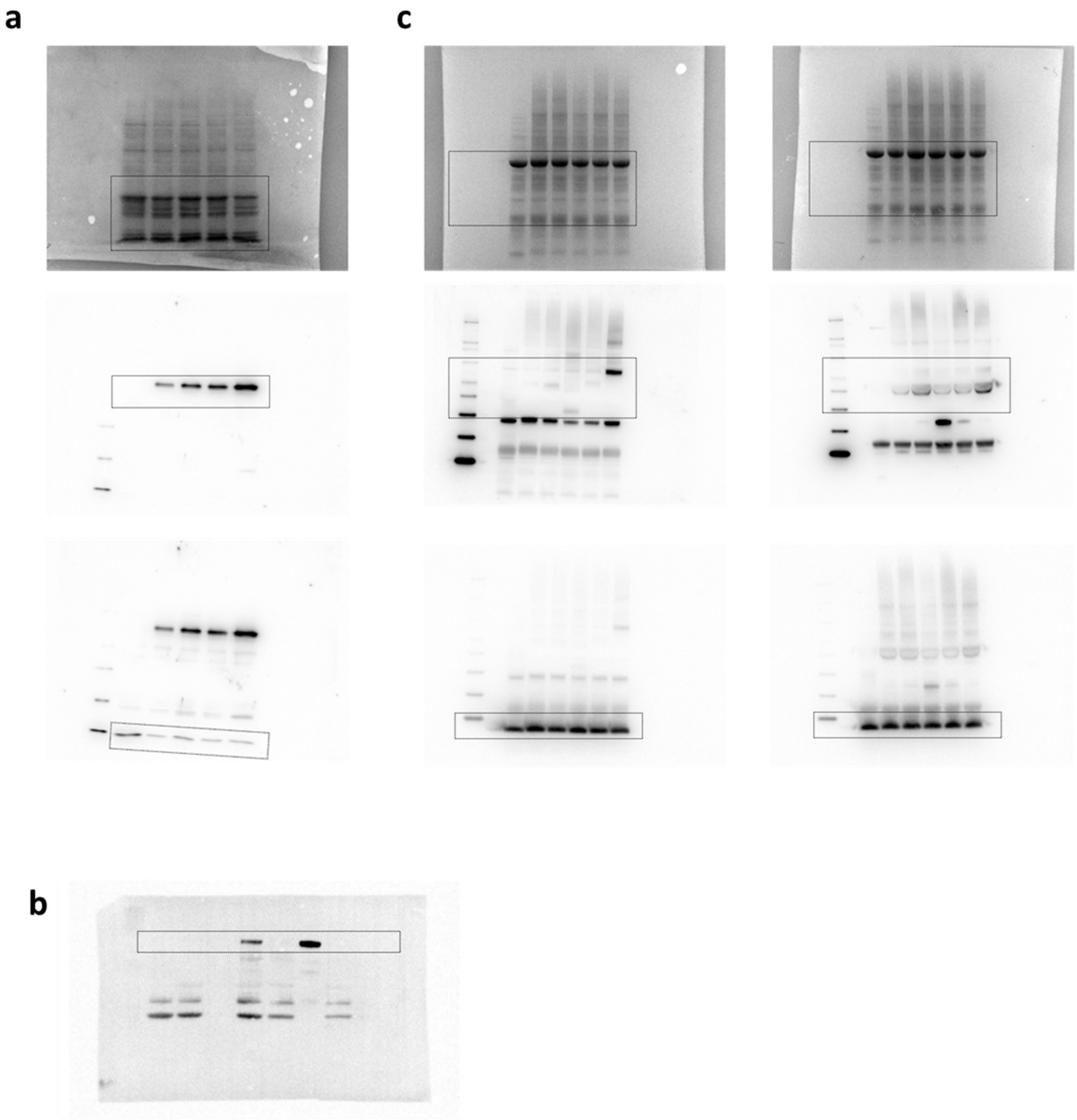

Supplementary Figure 9. Full scan images of Western blots. (**a**) Membrane from Fig. 1. (**b**) Membrane from Supplementary Fig. 1b. (**c**) Membranes from Supplementary Fig. 4. The parts of the membranes that are presented in the figures are boxed. The molecular weight ladder that was used is MagicMark™ XP Western Protein Standard (Invitrogen).

Supplementary Table 1. GO tagging of DOG1 interacting proteins involved in seed physiology, response to ABA, or protein dephosphorylation.

Gene ID	Annotation	Associated GO(s)
AT3G04120	Glyceraldehyde-3-Phosphate dehydrogenase C subunit 1 (GAPC1)	seed development (GO: 0048316)
AT5G46290	3-ketoacyl-acyl carrier protein synthase 1 (KAS1)	embryo development ending in seed dormancy (GO:0009793); seed development (GO: 0048316)
AT3G47520	MDH malate dehydrogenase (MDH)	embryo development ending in seed dormancy (GO:0009793); seed development (GO: 0048316)
AT5G37510	NADH-ubiquinone dehydrogenase, mitochondrial, putative	embryo development ending in seed dormancy (GO:0009793); seed development (GO: 0048316)
AT4G34200	D-3-phosphoglycerate dehydrogenase	embryo development ending in seed dormancy (GO:0009793); seed development (GO: 0048316)
AT3G53040	late embryogenesis abundant protein, putative / LEA protein, putative	embryo development ending in seed dormancy (GO:0009793); seed development (GO: 0048316)
AT2G42560	late embryogenesis abundant domain-containing protein / LEA domain-containing protein	embryo development ending in seed dormancy (GO:0009793); seed development (GO: 0048316)
AT2G39990	EIF2 Eukaryotic translation Initiation Factor 2 (EIF2)	embryo development ending in seed dormancy (GO:0009793); seed development (GO: 0048316)
AT1G74960	Fatty Acid Biosynthesis 1 (FAB1)	embryo development ending in seed dormancy (GO:0009793); seed development (GO: 0048316)
AT1G72100	late embryogenesis abundant domain-containing protein / LEA domain-containing protein	embryo development ending in seed dormancy (GO:0009793); seed development (GO: 0048316)
AT1G64520	Regulatory Particle Non-ATPase 12A (RPN12a)	embryo development ending in seed dormancy (GO:0009793); seed development (GO: 0048316)
AT1G34430	2-oxoacid dehydrogenases acyltransferase family protein	embryo development ending in seed dormancy (GO:0009793); seed development (GO: 0048316)
AT5G61780	TUDOR-SN protein 2 (TSN2)	seedling development (GO:0090341); seed germination (GO:0009845)
AT5G56030	Heat Shock Protein 81-2 (HSP81-2)	regulation of seedling development (GO:1900140); seedling development (GO:0090341); negative regulation of seed germination (GO:0010187); regulation of seed germination (GO:0010029); seed germination (GO:0009845); positive regulation of seed dormancy process (GO:1902040)
AT5G15090	Voltage Dependent Anion Channel 3 (VDAC3)	regulation of seedling development (GO:1900140); seedling development (GO:0090341); regulation of seed germination (GO:0010029); seed germination (GO:0009845); positive regulation of seed dormancy process (GO:1902040)
AT5G59310	Lipid Transfer Protein 4 (LTP4)	response to ABA (GO:0009737)
AT4G38970	Fructose-Bisphosphate Aldolase 2 (FBA2)	response to ABA (GO:0009737)
AT2G47770	Outer membrane tryptophan-rich sensory protein-related (TSPO)	response to ABA (GO:0009737)

Gene ID	Annotation	Associated GO(s)
AT1G54100	Aldehyde dehydrogenase 7B4 (ALDH7B4)	response to ABA (GO:0009737)
AT1G05510	Protein of unknown function (DUF1264)	response to ABA (GO:0009737); embryo development ending in seed dormancy (GO:0009793); seed oilbody biogenesis (GO:0010344); seed development (GO: 0048316)
AT3G25800	Protein phosphatase 2A subunit A2 (PP2AA2/PDF1)	protein dephosphorylation (GO:0006470)
AT5G51760	Protein phosphatase 2C family protein; ABA Hypersensitive Germination 1 (AHG1)	protein dephosphorylation (GO:0006470); response to ABA (GO:0009737)
AT3G11410	Protein phosphatase 2CA (PP2CA); ABA Hypersensitive Germination 3 (AHG3)	protein dephosphorylation (GO:0006470); response to ABA (GO:0009737)
AT4G11040	Protein phosphatase 2C family protein; Reduced Dormancy 5 (RDO5)	protein dephosphorylation (GO:0006470); regulation of seed dormancy process (GO:2000033) ; seed dormancy process (GO:0010162) ; positive regulation of seed maturation (GO:2000693); regulation of seed maturation (GO:2000034); seed maturation (GO:0010431); regulation of seed development (GO: 0080050); seed development (GO: 0048316)

Supplementary Table 2. Primers used in this study.

Name	Primer sequence 5’-3’	Purpose
Entr-YFP-F	CAC CAT GGT GAG CAA GGG CGA	Cloning of *pDOG1_Cvi:YFP:DOG1*$_{Cvi}$
mutNot-YFP-R	GCT GCT GCG GAG CCT GCT TTT TTG CTC TTG TAC AGC TCG TCC ATG	Cloning of *pDOG1_Cvi:YFP:DOG1*$_{Cvi}$
mutNot-entrDOG1-F	CCG CAG CAG CCC CCT TCA CCA TGG GAT CTT CAT CAA AGA A	Cloning of *pDOG1_Cvi:YFP:DOG1*$_{Cvi}$
DOG1-far down-R	TTT GGG GTC TAA ACC TTG CAT ATA TCA	Cloning of *pDOG1_Cvi:YFP:DOG1*$_{Cvi}$
Not-proDOG1-F	AAG CGG CCG CAC CAA ATT GTT TGT GCA TGC TTC AG	Cloning of *pDOG1_Cvi:YFP:DOG1*$_{Cvi}$
Not-proDOG1-R	TTG CGG CCG CGA TCT CTT TTG GTT TGC GTG TTT GTG	Cloning of *pDOG1_Cvi:YFP:DOG1*$_{Cvi}$
AHG1-noATG-F	AAA AAG CAG GCT ATA CTG AAA TCT ACA GAA CAA TTT C	Cloning of full length AHG1 CDS for Y2H
AHG1-stop-R	AGA AAG CTG GGT AAT ATC AAC ATC TTA TTC TTT GG	Cloning of full length AHG1 CDS for Y2H and recombinant protein
AHG3-noATG-F	AAA AAG CAG GCT ATG CTG GGA TTT GTT GC	Cloning of full length AHG3 CDS for Y2H
AHG3-stop-R	AGA AAG CTG GGT ACA CTA ATT ATT AAG ACG ACG	Cloning of full length AHG3 CDS for Y2H
AHG1_EF1	CAT TGT GCA GCA CGA CTA TGC	Characterization insertion mutants
AHG1_ER1	TGG TTC CCA TGC TAC CAT TGG	Characterization insertion mutants
AHG1_LP	ACC GAC ACG TGT TCT GTC TTC	Characterization insertion mutants
AHG1_RP	CTA AAA CTC GAC CAC CAG CTG	Characterization insertion mutants
AHG3_EF1	TTC TAC GGT GTC TTT GAC GGC	Characterization insertion mutants
AHG3_ER1	AAC TCC AAG AAC CCT AGC TCC	Characterization insertion mutants
AHG3_LP	TTT GGT TGA TTT TAG GTT GCG	Characterization insertion mutants
AHG3_RP	TTC CCC AGC CTG AAT TAA GAG	Characterization insertion mutants
PDF1_LP	GGA TCA TCA AGT TCC TTA AGC C	Characterization insertion mutants
PDF1_RP	TCA GAA GGC TTT CTA CGA TCG	Characterization insertion mutants

PDF1_GWR	C TTA GCT AGA CAT CAT CAC ATT GTC	Characterization insertion mutants
dog1-2_Mse_F	TTC TTT AGG CTC GTT TAT GCT TTG TGT GGT T	Identification of *dog1-2* mutation
dog1-2_Mse_R	CTG ACT ACC GAA CCA AAA AAT TGA ATT TAG TC	Identification of *dog1-2* mutation
SALK_left	CGA TTT CGG AAC CAC CAT CAA ACA GGA	Identification of SALK T-DNA insertion
PDF1-F	GGG GAC AAG TTT GTA CAA AAA AGC AGG CTT CGA AGG AGA TAG AAC CAT GTC TAT GAT CGA TGA GCC	Cloning of full length PDF1 CDS for Y2H
PDF1-R	GGG GAC CAC TTT GTA CAA GAA AGC TGG GTG GCT CTA CAA TCT ATT GAC AAT GTG ATG	Cloning of full lengh PDF1 CDS for Y2H
AHG1-F	GGG GAC AAG TTT GTA CAA AAA AGC AGG CTT CAT GAC TGA AAT CTA CAG AAC	Cloning of full length AHG1 CDS for BiFC
AHG1-nostop-R	GGG GAC CAC TTT GTA CAA GAA AGC TGG GTC CTG AGA GCT ATT CTT GAG AT	Cloning of full length AHG1 CDS for BiFC
AHG3-F	GGG GAC AAG TTT GTA CAA AAA AGC AGG CTT CAT GGC TGG GAT TTG TTG CGG	Cloning of full length AHG3 CDS for BiFC
AHG3-nostop-R	GGG GAC CAC TTT GTA CAA GAA AGC TGG GTC AGA CGA CGC TTG ATT ATT CC	Cloning of full length AHG3 CDS for BiFC
PDF1-F2	GGG GAC AAG TTT GTA CAA AAA AGC AGG CTT CAT GTC TAT GAT CGA TGA GCC	Cloning of full length PDF1 CDS for BiFC
PDF1-nostop-R	GGG GAC CAC TTT GTA CAA GAA AGC TGG GTC GCT AGA CAT CAT CAC ATT GT	Cloning of full length PDF1 CDS for BiFC
RDO5-F	GGG GAC AAG TTT GTA CAA AAA AGC AGG CTT CAT GAA AAC GGA TAC TAC TCT	Cloning of full length RDO5 CDS for BiFC
RDO5-nostop-R	GGG GAC CAC TTT GTA CAA GAA AGC TGG GTC AGA AAC GGT AGA GCT TTT GA	Cloning of full length RDO5 CDS for BiFC
ABI2-F	GGG GAC AAG TTT GTA CAA AAA AGC AGG CTT CAT GGA CGA AGT TTC TCC TGC	Cloning of full length ABI2 CDS for BiFC
ABI2-nostop-R	GGG GAC CAC TTT GTA CAA GAA AGC TGG GTC ATT CAA GGA TTT GCT CTT GA	Cloning of full length ABI2 CDS for BiFC
AHG1-noATG-TEV-F	AAA AAG CAG GCT ATG AAA ACC TGT ATT TTC AGG GCA CTG AAA TCT ACA GAA CAA TTT C	Cloning of full length AHG1 CDS for recombinant protein
LinkStrep-NcoI-F	AAA ACC ATG GCT AGC TGG AGC CAC CCG CAG TTC GAA AAA GAC GAC GAC GAC AAG GCT AGC	Synthetic Strep-tag linker reconstitution
LinkStrep-NcoI-R	GCT AGC CTT GTC GTC GTC GTC TTT TTC GAA CTG CGG GTG GCT CCA GCT AGC CAT GGT TTT	Synthetic Strep-tag linker reconstitution
DOG1-F	ATG GGA TCT TCA TCA AAG AAC ACC	Cloning of full length DOG1β CDS for recombinant protein
DOG1-BamHI-R	TTT TGG ATC CCT ACT TTC CTT CCT CTC C	Cloning of full length DOG1β CDS for recombinant protein
Att-adapter-F	GGG GAC AAG TTT GTA CAA AAA AGC AGG CT	Extension of Gateway AttB1 site
Att-adapter-R	GGG GAC CAC TTT GTA CAA GAA AGC TGG GT	Extension of Gateway AttB1 site

Supplementary reference:

Xiang, Y., Song, B., Née, G., Kramer, K., Finkemeier, I., & Soppe, W.J.J. Sequence polymorphisms at the REDUCED DORMANCY 5 pseudophosphatase underlie natural variation in Arabidopsis dormancy. *Plant Physiol.* **171**, 2659-2670 (2016).
